# Global, regional, and national burdens of pruritus in children and adolescents aged under 20 years from 1990 to 2021: a trend analysis

**DOI:** 10.3389/fmed.2025.1698461

**Published:** 2025-12-17

**Authors:** Xiangyun Ding, Zhi Yang, Haoye Lu, Bangtao Chen

**Affiliations:** 1 Department of Dermatology, Shenzhen Pingle Orthopedic Hospital (Shenzhen Pingshan Traditional Chinese Medicine Hospital), Shenzhen, China; 2 Department of Dermatology, The Third Affiliated Hospital of Chongqing Medical University, Chongqing, China; 3 Department of Dermatology, The Affiliated Hospital of North Sichuan Medical College, Nanchong, China; 4 Department of Dermatology, Chongqing University Three Gorges Hospital, School of Medicine, Chongqing University, Chongqing, China

**Keywords:** pruritus, children, adolescents, global burden of disease, trend analysis

## Abstract

**Objective:**

This study aims to analyze the global pruritus burden and trend among children and adolescents aged under 20 from 1990 to 2021 based on the existing Global Burden of Disease Study 2021 (GBD 2021) database.

**Methods:**

The incidence, prevalence, and disability-adjusted life-years (DALYs) of pruritus in children and adolescents were retrieved from the GBD 2021 database. Per 100,000 population, the age-standardized incidence rate (ASIR), age-standardized prevalence rate (ASPR), age-standardized DALY rate (ASDR), and corresponding estimated annual percent change (EAPC) were calculated in different dimensions, including the sociodemographic index (SDI), gender, age (<5, 5–9, 9–14, and 15–19 years), and region. Frontier, decomposition, and inequality analyses and projections of pruritus burden up to 2045 were conducted.

**Results:**

In 2021, 14899940.11 children and adolescents (including 12124676.85 new cases) suffered from pruritus, with an ASIR of 458.02, ASPR of 559.72, and ASDR of 6.09. Higher ASIR (510.85 vs. 408.33), ASPR (625.2 vs. 498.12), and ASDR (6.79 vs. 5.42) were found in females than in males, and they were projected to reach 525.47, 673.99, and 7.32 for females and 418.52, 535.95, and 5.86 for males in 2045. ASIR (411.47 to 526.3), ASPR (397.89 to 681.96), and ASDR (4.33 to 7.4) were all gradually increasing from the group with age younger than 5 years to the group with 15–19 years. Low SDI regions had the highest ASIR (493.99), ASPR (614.24), and ASDR (6.66). Eastern/Southern/Western Sub-Saharan Africa and the United Republic of Tanzania had the heaviest pruritus burden, whereas Germany had the lightest. Globally, EAPC showed annual increases of 0.32 for ASIR, 0.35 for ASPR, and 0.36 for ASDR; the most rapid escalation in ASIR was found in East Asia, ASPR and ASDR in Australasia among regions, and ASIR, ASPR, and ASDR were found in the Netherlands among countries.

**Conclusion:**

The overall pruritus burden in children and adolescents has risen substantially from 1990 to 2021 and has increased with age, with females and individuals in low-SDI regions being more affected. The burden may continue to increase with population growth over the next 20 years. Further strengthening policies tackling burdensome pruritus is warranted, especially in East Asia, Australasia, and the Netherlands, where the pruritus burden in children and adolescents increases rapidly.

## 1 Introduction

Pruritus symptoms theoretically occur in dermatosis and other diseases, including kidney, hepatobiliary, metabolic, endocrine, malignancy, infectious, neurological, and psychiatric disorders (1, 2). Therefore, the etiologies, pathophysiological mechanisms, and related treatment strategies for pruritus may vary depending on the disease types. Similar to the management of pain symptoms, pruritus clinics have emerged in most tertiary hospitals in China. The emergence of precision medicine encourages dermatologists to focus on comprehensive clinical management of pruritus (3, 4). To refine the management of pruritus, the collection of epidemiological data in specific populations is particularly important, as it provides critical support for health management departments making health decisions (including the directions for research funding and drug development).

Among the recorded pruritic diseases, some occur at all ages but have specific characteristics during adolescence (such as atopic dermatitis), and some mainly occur in teenagers (such as acne). Pruritus seriously affects sleep quality and learning efficiency and even causes psychiatric disorders, which are greatly detrimental to long-term career planning in children and adolescents (5). In addition, once teenagers suffer from pruritic diseases, their guardians also show great concern, and thus, work efficiency is impaired. Wang et al. (6) and Jin et al. (7) recently revealed the burden of the pruritus symptom of the entire population at the global, regional, and national levels based on the Global Burden of Disease Study 2021 (GBD 2021) database; however, they did not perform decomposition, cross-country inequality, frontier, and predictive analyses in children and adolescents, and the pruritus burden in the whole children and adolescents was also not calculated. To address the global public health challenge in children and adolescents posed by pruritus more effectively and inform healthcare decision-makers implementing reasonable policies, this study focused on children and adolescents aged under 20 years and systematically performed analyses of pruritus incidence, prevalence, disability-adjusted life-years (DALYs), and trends using the GBD 2021 database.

## 2 Materials and methods

### 2.1 Data source

Data on pruritus from 1990 to 2021 were retrieved from the GBD 2021 database, which is accessible through the GBD results tool on the Institute for Health Metrics and Evaluation (IHME) website.^1^ The GBD study 2021 is a systematic survey evaluating the health impacts of diseases, injuries, and risk factors stratified by several dimensions, including age, sex, and region. We performed a systematic analysis of the incidence, prevalence, and DALYs associated with pruritus among children and adolescents aged under 20 years, examining these metrics at the global, regional, and national levels from 1990 to 2021. DALYs, which are quantified as the aggregate of years of life lost (YLLs) and years lived with disability (YLDs), serve as a comprehensive metric for assessing the overall burden of pruritus (8). Additionally, to determine the development across countries and territories (9), we introduced the sociodemographic index (SDI), which reflects the comprehensive level of income, education, and fertility levels. According to the SDI level and quintile rankings, the locations were categorized into five groups: low, low-middle, middle, high-middle, and high.

### 2.2 Statistical analysis

Employing standardized GBD methodologies, we aimed to derive a more precise representation of the health burden attributable to pruritus by integrating distinct types of epidemiological data, namely the incidence, prevalence, and DALYs. The presented data were meticulously estimated and displayed both as numerical counts per 100,000 population and as age-standardized rates (ASRs), accompanied by uncertainty intervals (UIs), to highlight potential statistical variability. Specifically, the age-standardized incidence rate, age-standardized prevalence rate, and age-standardized DALY rate are abbreviated as ASIR, ASPR, and ASDR, respectively.

The estimated annual percent change (EAPC), a consolidated overview of trends across the study period, was also computed. A linear regression model was first constructed as y = *α* + *β*x, where y = ln (ASR) and x = calendar year. Then, the EAPC was calculated by (exp(β)−1) * 100%, and its 95% confidence interval (CI) was also derived from the model (10). An upward trend of ASR is defined as both the EAPC value and its lower boundary of 95% CI > zero; a downward trend of ASR is defined as both the EAPC value and its upper boundary of 95% CI < zero; a stable trend of ASR is recognized in the other conditions.

To recognize the inflection points (termed “joinpoints”) in disease trends, a joinpoint regression model was also applied, which can discriminate notable changes in epidemiological patterns. Between each pair of adjacent joinpoints, the model calculates the annual percent change (APC) in rates, quantifying the magnitude of the trend within that segment. Additionally, we calculated the average annual percent change (AAPC) across the entire study period. AAPC represents a weighted average of all segment-specific APCs, providing an integrated measure of the overall trend progression.

To elucidate the separate contribution of population age structure, population growth, and epidemiological alterations to the health burden of pruritus, decomposition analysis was carried out (11). This analysis entails assessing the contribution of each factor independently while holding the other two factors fixed, thereby determining the extent to which epidemiological trends are affected by each specific factor.

We further examined health inequalities using two standardized metrics: the inequality slope index (absolute inequality) and the concentration index (relative inequality). The inequality slope index was derived via regression, linking country-level DALYs to their SDI position—ranked by cumulative population distribution (12). The concentration index was calculated by plotting a Lorenz concentration curve and comparing the cumulative distribution of the SDI-ranked populations against their corresponding DALYs, followed by numerical integration of the area under the curve.

Frontier analysis was performed to evaluate the potential improvement space for the burden of pruritus across 204 countries and territories, utilizing data spanning from 1990 to 2021. This analysis employs age-standardized DALYs alongside the SDI to assess the scope for improvement at the corresponding SDI levels (13).

Future trends through 2045 were projected using the Bayesian Age-Period-Cohort (BAPC) model under the Integrated Nested Laplace Approximations (INLA) framework, chosen for its computational efficiency and reduced error rates compared to conventional methods (14). This model builds on the traditional generalized linear model framework within a Bayesian context, allowing the dynamic integration of age, period, and cohort effects. These effects are assumed to evolve continuously over time and are smoothed using a second-order random walk, resulting in more accurate posterior probability predictions. The main operational steps include inputting the data, smoothing priorities, and forecasting mechanisms.

All the above-mentioned procedures for analysis and graphic representation were performed utilizing the statistical computing software, R (Version 4.3.2).

## 3 Results

### 3.1 Global trends

Among children and adolescents aged under 20 years, the global number of newly reported cases of pruritus was documented as 12124676.85 (95% UI, 8639449.31 to 16262561.04) in 2021 (Supplementary Table 1), with an ASIR of 458.02 per 100,000 population (Table 1). The ASPR and ASDR were recorded as 559.72 (95% UI, 401.64 to 753.04) and 6.09 (95% UI, 2.72 to 11.97), respectively. EAPC analysis indicated a significant increase in the global burden of pruritus from 1990 to 2021, as evidenced by the EAPC’s estimate alongside its 95% CI lower bound exceeding zero (Table 2).

**Table 1:** The burden of pruritus (ASIR, ASPR, and ASDR) globally and by sex, age group, region, and SDI quintile in 2021 (per 100,000 population).

Items	ASIR (95% UI)	ASPR (95% UI)	ASDR (95% UI)
Global	458.02 (327.15 to 613.15)	559.72 (401.64 to 753.04)	6.09 (2.72 to 11.97)
Sex			
Female	510.85 (365.13 to 683.36)	625.2 (448.88 to 841.21)	6.79 (3.04 to 13.36)
Male	408.33 (291.35 to 547.01)	498.12 (357.52 to 670.18)	5.42 (2.42 to 10.67)
Age groups (years)			
<5	411.47 (318.86 to 515.49)	397.89 (315.42 to 494.76)	4.33 (2.08 to 8.05)
5–9	439.67 (307.42 to 592.49)	575.29 (408.29 to 749.48)	6.25 (2.86 to 12.05)
10–14	466.79 (324.16 to 640.39)	611.02 (428.68 to 843.88)	6.65 (2.87 to 13.05)
15–19	526.3 (363.43 to 725.98)	681.96 (468.99 to 971.52)	7.4 (3.18 to 15.43)
SDI category			
High SDI	379.81 (284.55 to 487.51)	461.05 (358.02 to 584.65)	5.03 (2.31 to 9.68)
High-middle SDI	462.41 (327.01 to 623.5)	565.73 (402.35 to 767.95)	6.17 (2.75 to 12.1)
Middle SDI	472.16 (335.38 to 634.92)	574.78 (411.29 to 775.85)	6.26 (2.78 to 12.34)
Low-middle SDI	439.9 (312.73 to 590.34)	534.15 (380.45 to 722.22)	5.8 (2.59 to 11.45)
Low SDI	493.99 (349.98 to 664.37)	614.24 (437.01 to 832.15)	6.66 (2.98 to 13.11)
Region			
Andean Latin America	392.01 (279.3 to 526.91)	472.21 (337.51 to 641.88)	5.13 (2.26 to 10.13)
Australasia	340.19 (240.41 to 457.19)	415.49 (295.59 to 567.16)	4.53 (1.93 to 9.05)
Caribbean	382.58 (271.45 to 512.4)	456.58 (326.35 to 622.82)	4.96 (2.23 to 9.77)
Central Asia	403.85 (286.83 to 543.54)	486.48 (346.84 to 660.83)	5.29 (2.35 to 10.39)
Central Europe	434.13 (309.52 to 582.6)	527.58 (375.27 to 712.56)	5.75 (2.54 to 11.29)
Central Latin America	412.33 (292.92 to 553.13)	498.63 (354.85 to 673.58)	5.43 (2.43 to 10.71)
Central Sub-Saharan Africa	476.87 (336.83 to 646.47)	582.3 (411.55 to 792.61)	6.32 (2.78 to 12.32)
East Asia	511.86 (362.24 to 691.93)	624.76 (444.38 to 848.46)	6.82 (3.03 to 13.43)
Eastern Europe	445.65 (315.84 to 600.54)	542.4 (386.04 to 733.55)	5.91 (2.62 to 11.6)
Eastern Sub-Saharan Africa	581.94 (410.4 to 786.12)	754.48 (533.26 to 1028.25)	8.19 (3.63 to 16.07)
High-income Asia Pacific	351.23 (249.32 to 471.76)	429.23 (305.7 to 581.76)	4.68 (2.09 to 9.23)
High-income North America	389.57 (305.06 to 478.16)	470.13 (396.74 to 551.88)	5.13 (2.4 to 9.78)
North Africa and the Middle East	442.26 (318.77 to 587.62)	532.39 (383.49 to 711.41)	5.79 (2.58 to 11.36)
Oceania	446.72 (316.21 to 599.41)	533.81 (376.17 to 725.07)	5.79 (2.54 to 11.42)
South Asia	405.09 (288.71 to 543.51)	487.87 (348.01 to 658.92)	5.29 (2.36 to 10.49)
Southeast Asia	505.8 (357.37 to 684.16)	614.39 (437.36 to 834.17)	6.69 (2.97 to 13.23)
Southern Latin America	322.01 (227.51 to 433.69)	391.25 (280.1 to 529.07)	4.26 (1.87 to 8.34)
Southern Sub-Saharan Africa	538.71 (382.21 to 724.82)	668.03 (474.92 to 911.03)	7.26 (3.23 to 14.24)
Tropical Latin America	421.39 (299.79 to 566.29)	511.18 (363.28 to 690.38)	5.55 (2.49 to 10.99)
Western Europe	317.89 (231.07 to 419.61)	382.74 (283.53 to 503.34)	4.17 (1.88 to 8.04)
Western Sub-Saharan Africa	526.27 (371.68 to 710.45)	653.99 (463.97 to 891.91)	7.09 (3.16 to 14.04)

**Table 2:** The estimated annual percent change (EAPC) of ASIR, ASPR, and ASDR attributable to pruritus globally and by sex, region, and SDI quintile from 1990 to 2021.

Items	ASIR (95% CI)	ASPR (95% CI)	ASDR (95% CI)
Global	0.32 (0.31 to 0.34)	0.35 (0.33 to 0.37)	0.36 (0.34 to 0.38)
Sex			
Female	0.31 (0.3 to 0.32)	0.34 (0.32 to 0.36)	0.34 (0.33 to 0.36)
Male	0.34 (0.33 to 0.36)	0.37 (0.35 to 0.39)	0.38 (0.36 to 0.4)
SDI category			
High SDI	0.23 (0.2 to 0.25)	0.24 (0.21 to 0.26)	0.24 (0.21 to 0.26)
High-middle SDI	0.3 (0.29 to 0.31)	0.33 (0.31 to 0.34)	0.33 (0.31 to 0.34)
Middle SDI	0.3 (0.29 to 0.32)	0.33 (0.32 to 0.35)	0.33 (0.32 to 0.35)
Low-middle SDI	0.35 (0.33 to 0.37)	0.39 (0.36 to 0.41)	0.39 (0.36 to 0.41)
Low SDI	0.22 (0.19 to 0.24)	0.25 (0.21 to 0.28)	0.33 (0.32 to 0.35)
Region			
Andean Latin America	0.17 (0.15 to 0.19)	0.14 (0.13 to 0.14)	0.14 (0.13 to 0.15)
Australasia	0.2 (0.17 to 0.22)	0.49 (0.47 to 0.51)	0.5 (0.48 to 0.51)
Caribbean	0.06 (0.06 to 0.07)	0.34 (0.33 to 0.36)	0.35 (0.34 to 0.37)
Central Asia	0.17 (0.1 to 0.23)	0.28 (0.22 to 0.34)	0.28 (0.22 to 0.34)
Central Europe	0.23 (0.2 to 0.26)	0.1 (0.09 to 0.11)	0.11 (0.1 to 0.11)
Central Latin America	0.1 (0.09 to 0.1)	0.23 (0.2 to 0.26)	0.23 (0.2 to 0.26)
Central Sub-Saharan Africa	0.04 (−0.01 to 0.09)	0.16 (0.1 to 0.23)	0.17 (0.1 to 0.24)
East Asia	0.46 (0.45 to 0.47)	0.19 (0.16 to 0.22)	0.2 (0.17 to 0.23)
Eastern Europe	0.16 (0.1 to 0.22)	0.23 (0.21 to 0.25)	0.24 (0.22 to 0.26)
Eastern Sub-Saharan Africa	0.15 (0.12 to 0.18)	0.24 (0.22 to 0.26)	0.24 (0.22 to 0.26)
High-income Asia Pacific	0.21 (0.19 to 0.23)	0.15 (0.08 to 0.22)	0.15 (0.08 to 0.22)
High-income North America	0.26 (0.2 to 0.32)	0.33 (0.31 to 0.35)	0.34 (0.32 to 0.35)
North Africa and the Middle East	0.23 (0.21 to 0.25)	0.05 (0.05 to 0.06)	0.05 (0.04 to 0.06)
Oceania	0.11 (0.09 to 0.12)	0.18 (0.15 to 0.21)	0.19 (0.16 to 0.22)
South Asia	0.34 (0.32 to 0.35)	0.21 (0.19 to 0.23)	0.21 (0.19 to 0.23)
Southeast Asia	0.3 (0.28 to 0.32)	0.04 (−0.02 to 0.1)	0.05 (−0.01 to 0.11)
Southern Latin America	0.22 (0.18 to 0.25)	0.09 (0.07 to 0.11)	0.1 (0.08 to 0.12)
Southern Sub-Saharan Africa	0.08 (0.07 to 0.09)	0.25 (0.23 to 0.26)	0.25 (0.23 to 0.26)
Tropical Latin America	0.14 (0.13 to 0.14)	0.19 (0.17 to 0.21)	0.2 (0.18 to 0.22)
Western Europe	0.23 (0.2 to 0.25)	0.24 (0.22 to 0.26)	0.25 (0.23 to 0.27)
Western Sub-Saharan Africa	0.16 (0.13 to 0.19)	0.08 (0.07 to 0.09)	0.09 (0.08 to 0.1)

#### 3.1.1 Global trends by sex

There were disparities between the two sexes, with higher numbers (Supplementary Table 1) and rates observed in females. Specifically, female children and adolescents had higher ASIR [510.85 (95% UI, 365.13 to 683.36)], ASPR [625.2 (95% UI, 448.88 to 841.21)], and ASDR [6.79 (95% UI, 3.04 to 13.36)] of pruritus than male children and adolescents (ASIR 408.33, ASPR 498.12, and ASDR 5.42) (Table 1). The global burden of pruritus among children and adolescents aged under 20 years demonstrated a consistent increase for both sexes from 1990 to 2021. Furthermore, the gender gap in these numbers and rates had slightly narrowed over the past decades, primarily due to a more pronounced increase among male children and adolescents, as indicated by the higher EAPCs in males (incidence, 0.34 vs. 0.31; prevalence, 0.37 vs. 0.34; DALYs, 0.38 vs. 0.34) than in females (Table 2).

#### 3.1.2 Global trends by age subgroup

Subgroup analysis was also conducted according to different age stages (<5, 5–9, 9–14, and 15–19 years old). In 2021, the peak ASIR, ASPR, and ASDR of pruritus were in those aged 15–19 years (Table 1), presenting as 526.3 (95% UI, 363.43 to 725.98), 681.96 (95% UI, 468.99 to 971.52), and 7.4 (95% UI, 3.18 to 15.43), respectively. Additionally, the lowest ASIR, ASPR, and ASDR of pruritus were in the <5 years group, recorded as 411.47 (95% UI, 318.86 to 515.49), 397.89 (95% UI, 315.42 to 494.76), and 4.33 (95% UI, 2.08 to 8.05), respectively. ASIR, ASPR, and ASDR of pruritus gradually increased as age increased.

#### 3.1.3 Global trends by SDI quintiles

In 2021, countries with a low SDI exhibited the highest ASIR, ASPR, and ASDR due to pruritus, with rates of 493.99 per 100,000 population (95% UI, 349.98 to 664.37), 614.24 per 100,000 population (95% UI, 437.01 to 832.15), and 6.66 per 100,000 population (95% UI, 2.98 to 13.11), respectively. These rates in low SDI countries were more than 1.3 times higher than those recorded in countries with a high SDI (Table 1). From 1990 to 2021, the pruritus burden (reflected by ASIR, ASPR, and ASDR) gradually increased in countries with different levels of SDI (Table 2).

#### 3.1.4 Regional trends

Regarding pruritus-related burden among the 21 regions, as shown in Table 1, Eastern Sub-Saharan Africa reported the highest ASIR (581.94 [95% UI, 410.4 to 786.12]), ASPR (754.48 [95% UI, 533.26 to 1028.25]), and ASDR (8.19 [95% UI, 3.63 to 16.07]), followed by Southern Sub-Saharan Africa (ASIR 538.71, ASPR 668.03, ASDR 7.26), Western Sub-Saharan Africa (ASIR 526.27, ASPR 653.99, ASDR 7.09), East Asia (ASIR 511.86, ASPR 624.76, ASDR 6.82), and Southeast Asia (ASIR 505.8, ASPR 614.39, ASDR 6.69). These rates significantly surpassed the global average (ASIR 458.02, ASPR 559.72, and ASDR 6.09). From 1990 to 2021, most regions experienced an increase in disease burden indicators for pruritus among children and adolescents, albeit at varying rates (Table 2). Notably, East Asia demonstrated the most rapid escalation in incidence (with EAPCs of 0.46%), followed by South Asia (with EAPCs of 0.34%) and Southeast Asia (with EAPCs of 0.30%). Australasia experienced the most significant increase in prevalence and DALYs attributable to pruritus, with an average annual trend of about 0.5%, followed by the Caribbean and high-income North America.

#### 3.1.5 National trends

**Figure 1:**
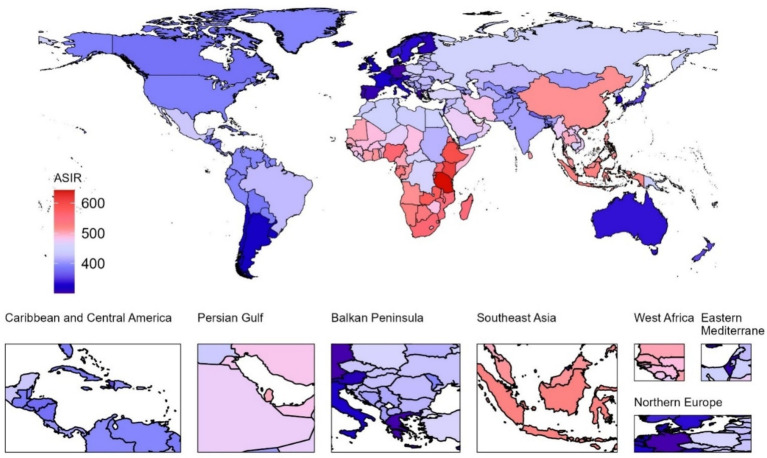
Age-standardized incidence rate (ASIR) associated with pruritus in children and adolescents across 204 countries and territories in 2021.

**Figure 2:**
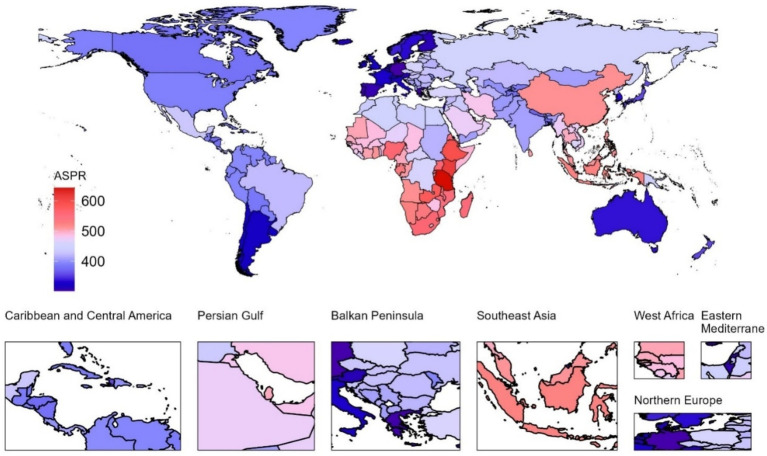
Age-standardized prevalence rate (ASPR) associated with pruritus in children and adolescents across 204 countries and territories in 2021.

**Figure 3:**
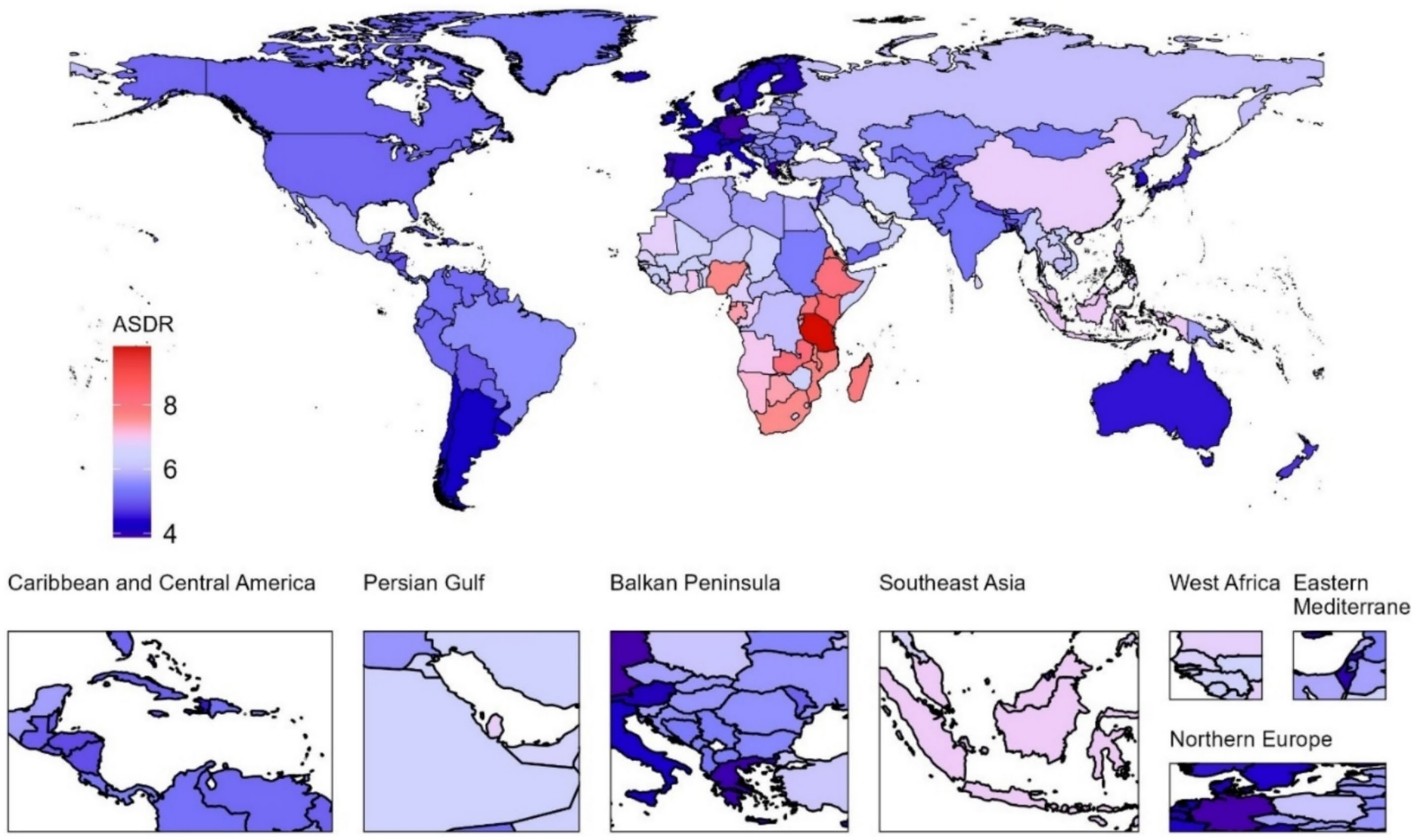
Age-standardized DALY rate (ASDR) associated with pruritus in children and adolescents across 204 countries and territories in 2021.

At the national level in 2021, the United Republic of Tanzania had the highest pruritus ASIR (Figure 1), ASPR (Figure 2), and ASDR (Figure 3) among children and adolescents, registering 643.26 (95% UI, 455.83 to 872.17), 905.1 (95% UI, 636.34 to 1235.3), and 9.81 (95% UI, 4.29 to 19.45), respectively; Kenya, Ethiopia, Zambia, and Djibouti, while Germany had the lowest ASIR [301.66 (95% UI, 228.98 to 384.5)], ASPR [356.43 (95% UI, 287.44 to 443.17)], and ASDR [3.88 (95% UI, 1.74 to 7.43)] of pruritus among children and adolescents. Notable variations in the burden of pruritus were observed across 204 countries (Supplementary Table 2). Between 1990 and 2021, a greater number of countries exhibited increasing trends in the burden of pruritus, except Barbados, Burundi, the Central African Republic, the Democratic People’s Republic of Korea, the Democratic Republic of the Congo, Haiti, Kyrgyzstan, Libya, Nauru, the Northern Mariana Islands, Somalia, South Sudan, Tajikistan, Ukraine, the United Arab Emirates, and Zimbabwe. During the observation period, the Netherlands exhibited the most significant increases in ASIR (average annual trends 0.66%), ASPR (average annual trends 0.83%), and ASDR (average annual trends 0.83%) related to pruritus, followed by Equatorial Guinea, China, Myanmar, and Bosnia and Herzegovina (Supplementary Table 3).

### 3.2 Decomposition analysis

**Figure 4:**
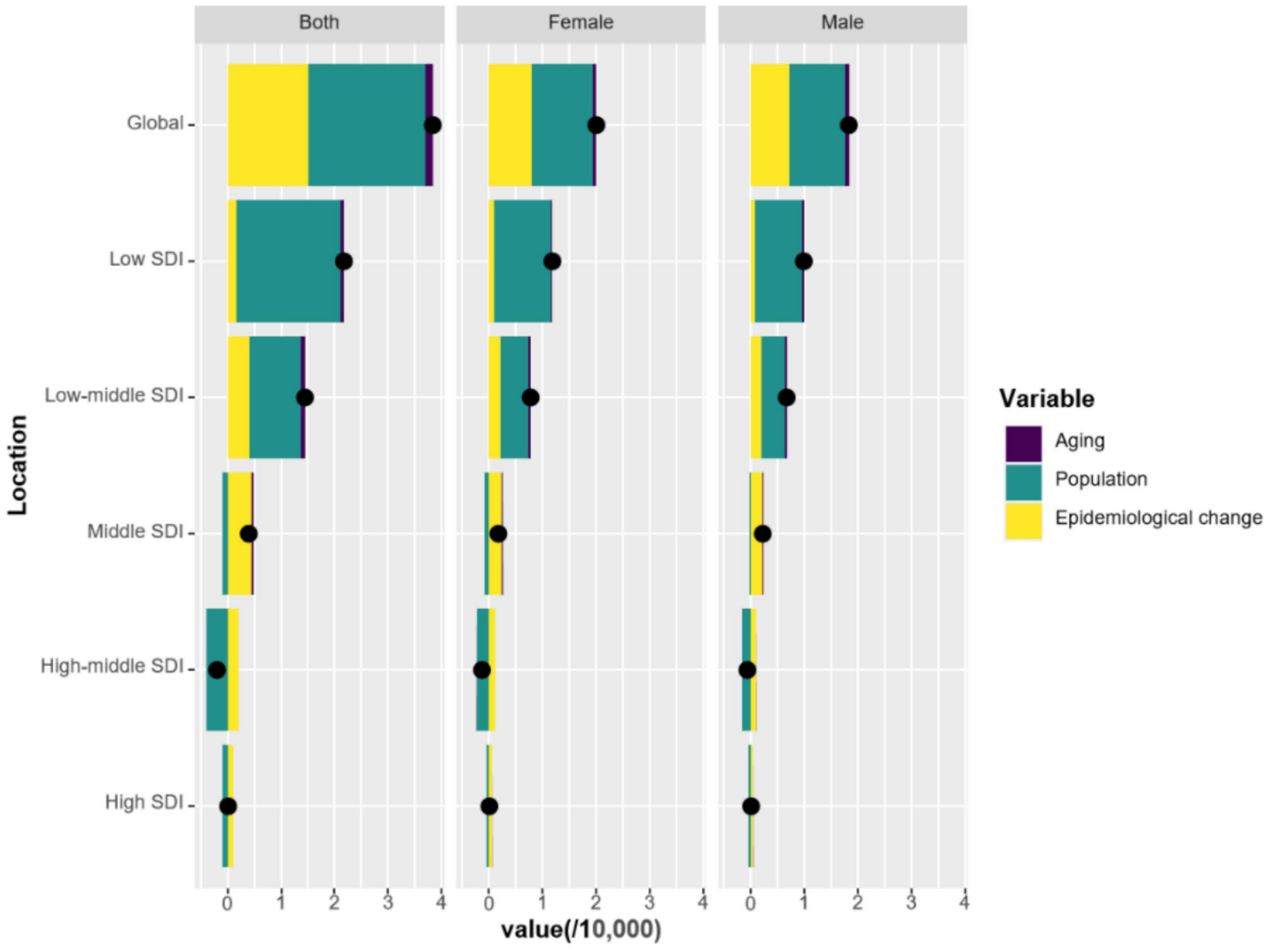
Changes in DALYs associated with pruritus according to population-level determinants of aging, population growth, and epidemiological change from 1990 to 2021 at the global level and by SDI quintile.

To evaluate the extent of aging, population growth, and epidemiological changes that have shaped the epidemiology of pruritus between 1990 and 2021, decomposition analyses were conducted in female and/or male children and adolescents. The findings revealed that population growth and epidemiological changes, but not aging, emerged as key contributors to the rising burden of pruritus (Figure 4). Specifically, this effect was most pronounced in the low SDI quintile, where population growth, epidemiological changes, and aging accounted for 88.68, 8.14, and 3.18% increases in pruritus-related DALYs, respectively (Table 3). Subgroup analysis by gender showed that population growth, epidemiological changes, and aging accounted for 89.13, 7.81, and 3.06% increase in female children and adolescents, and for 88.19, 8.51, and 3.31% increase in male children and adolescents, respectively, which was almost the same as the global analysis.

**Table 3:** Decomposition analysis of trends in ASDR for pruritus from 1990 to 2021, categorized by sex, global, and SDI regions.

Sex	Location	Overall difference	Aging	Population growth	Epidemiological change
Both	Global	38351.17	1416.92 (3.69%)	21916.45 (57.15%)	15017.8 (39.16%)
	High SDI	116.39	16.29 (14%)	−906.26 (−778.68%)	1006.36 (864.68%)
	High-middle SDI	−1954.6	28.12 (−1.44%)	−3981.26 (203.69%)	1998.54 (−102.25%)
	Middle SDI	3974.16	391.75 (9.86%)	−926.78 (−23.32%)	4509.18 (113.46%)
	Low-middle SDI	14464.85	813.38 (5.62%)	9525.33 (65.85%)	4126.14 (28.53%)
	Low SDI	21739.36	691.12 (3.18%)	19278.09 (88.68%)	1770.14 (8.14%)
Female	Global	20021.58	712.5 (3.56%)	11444.56 (57.16%)	7864.51 (39.28%)
	High SDI	48.22	11.93 (24.74%)	−525.11 (−1088.94%)	561.4 (1164.21%)
	High-middle SDI	−1331.76	−2.04 (0.15%)	−2364.46 (177.54%)	1034.74 (−77.7%)
	Middle SDI	1729.73	173.85 (10.05%)	−844.57 (−48.83%)	2400.45 (138.78%)
	Low-middle SDI	7749.1	419.53 (5.41%)	5145.01 (66.39%)	2184.56 (28.19%)
	Low SDI	11821.27	362.32 (3.06%)	10,536 (89.13)	922.95 (7.81%)
Male	Global	18329.59	694.28 (3.79%)	10375.19 (56.6%)	7260.12 (39.61%)
	High SDI	68.16	0.92 (1.34%)	−384.91 (−564.69%)	452.16 (663.35%)
	High-middle SDI	−622.84	26.76 (−4.3%)	−1662.34 (266.9%)	1012.74 (−162.6%)
	Middle SDI	2244.43	216.23 (9.63%)	−148.46 (−6.61%)	2176.66 (96.98%)
	Low-middle SDI	6715.75	391.16 (5.82%)	4366.37 (65.02%)	1958.22 (29.16%)
	Low SDI	9918.09	328.06 (3.31%)	8746.48 (88.19%)	843.55 (8.51%)

### 3.3 Cross-country inequality analysis

**Figure 5:**
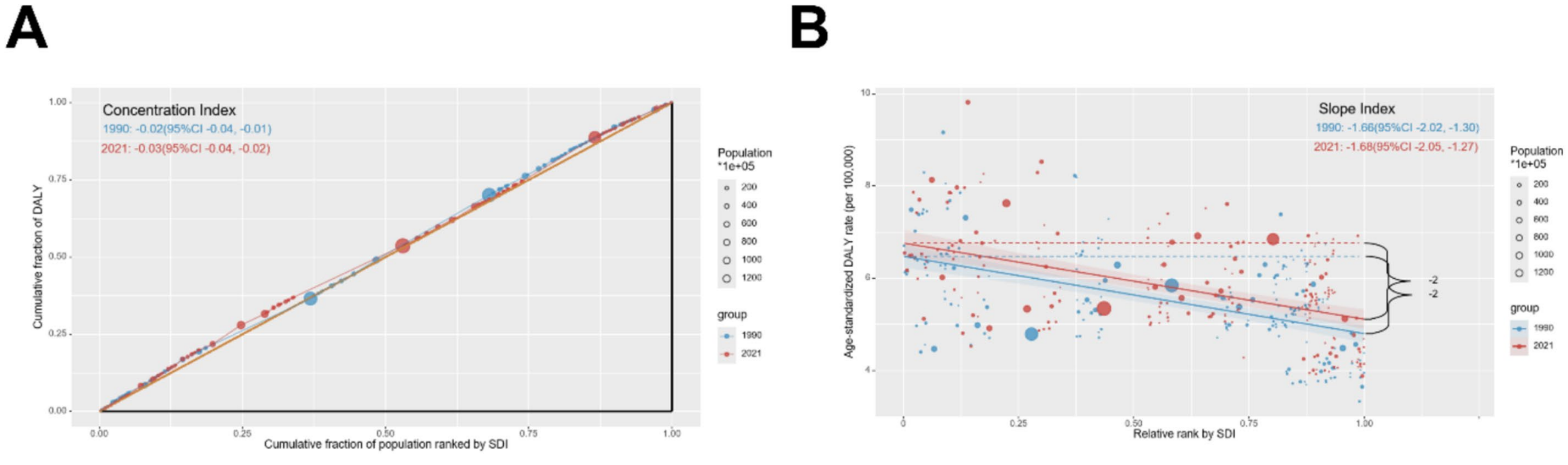
Inequality **(A)** concentration index and **(B)** slope index for pruritus DALYs worldwide from 1990 to 2021.

The quantification of cross-country SDI-related inequalities in the burden of pruritus was employed to elucidate the distribution pattern of pruritus burden across varying levels of sociodemographic development. The concentration index indicated a slight decrease from 1990 to 2021, suggesting slightly narrowed inequalities (Figure 5A). Significant absolute and relative SDI-related inequalities were identified, with a disproportionately greater burden shouldered by countries with a lower SDI (Figure 5B). As illustrated by the slope index of inequality, the gap of DALYs attributable to pruritus between countries with the highest and lowest SDI decreased from −1.66 (95% CI: −2.02 to −1.30) in 1990 to −1.68 (95% CI: −2.05 to −1.27) in 2021, which means that there was an excess of 1.66 (per 100,000 population) DALYs in countries with the lowest SDI compared to that in countries with the highest SDI in 1990, and this gap further enlarged to 1.68 in 2021.

### 3.4 Frontier analysis for the association between ideal pruritus DALYs and SDI

**Figure 6:**
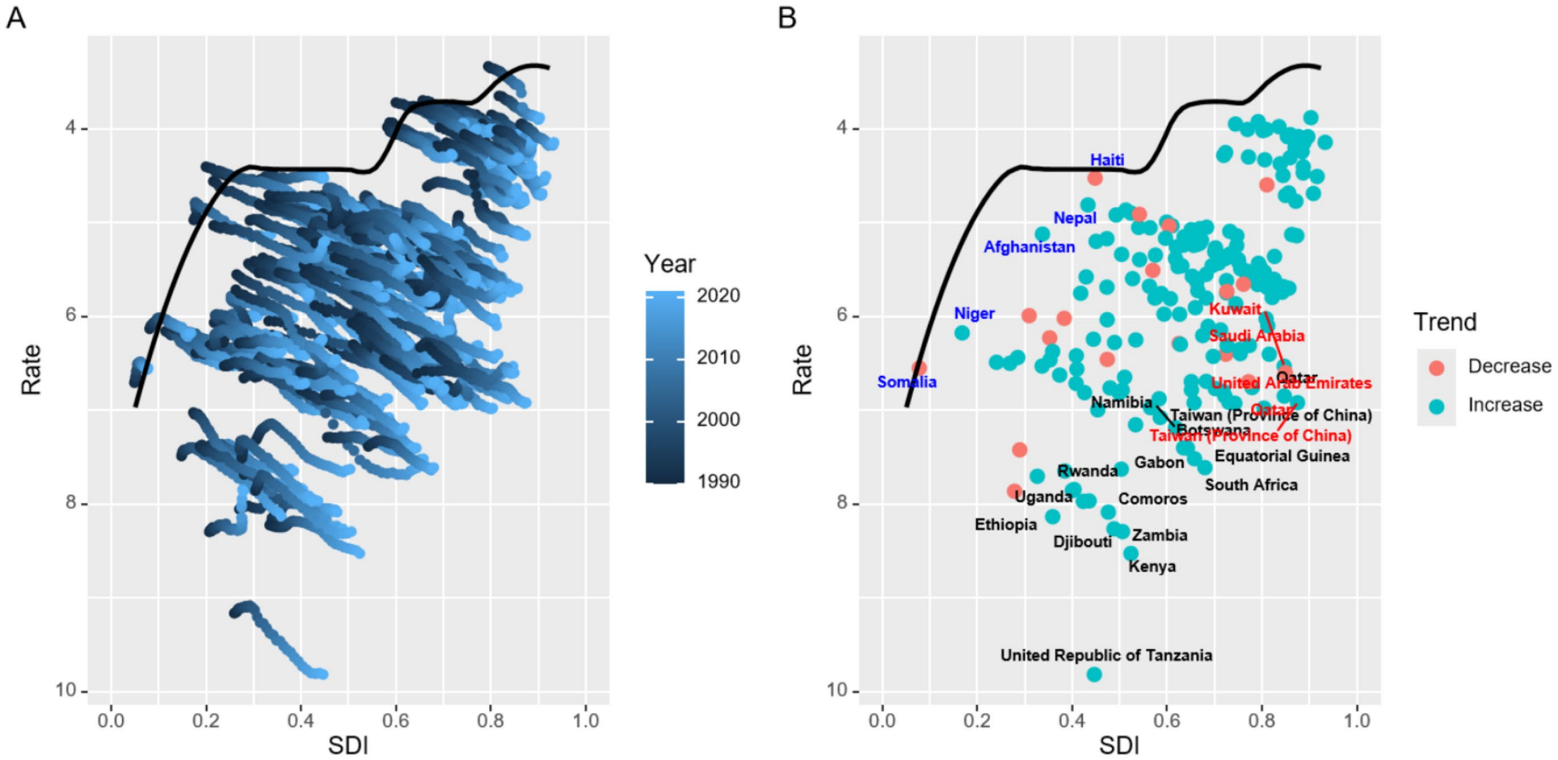
Frontier analysis based on age-standardized DALY rates of pruritus from 1990 to 2021.

Frontier analysis was performed to evaluate the associations among ideal pruritus DALYs, SDI, and effective difference (ED). Results showed that the five countries with the lower SDI [Haiti (SDI 0.45, ED 0.09), Nepal (SDI 0.43, ED 0.38), Afghanistan (SDI 0.34, ED 0.69), Niger (SDI 0.17, ED 0.16), Somalia (SDI 0.08, ED 0.07)] indicated in blue were closest to the frontier fit line, the five countries with the higher SDI [Kuwait (SDI 0.85, ED 3.18), Saudi Arabia (SDI 0.82, ED 3.05), United Arab Emirates (SDI 0.85, ED 3.24), Qatar (SDI 0.85, ED 3.49), and Taiwan/Province of China (SDI 0.87, ED 3.56)] highlighted in red were furthest from the frontier fit line, and there were 15 countries (marked in black) furthest from the frontier fit line in all countries regardless of SDI (Figure 6). In terms of DALYs attributable to pruritus, the countries and regions with the highest SDI furthest from the frontier line also exhibited significantly higher rates of pruritus DALYs compared to other countries with comparable sociodemographic profiles (Supplementary Table 4).

### 3.5 Projections of pruritus up to 2045

**Figure 7:**
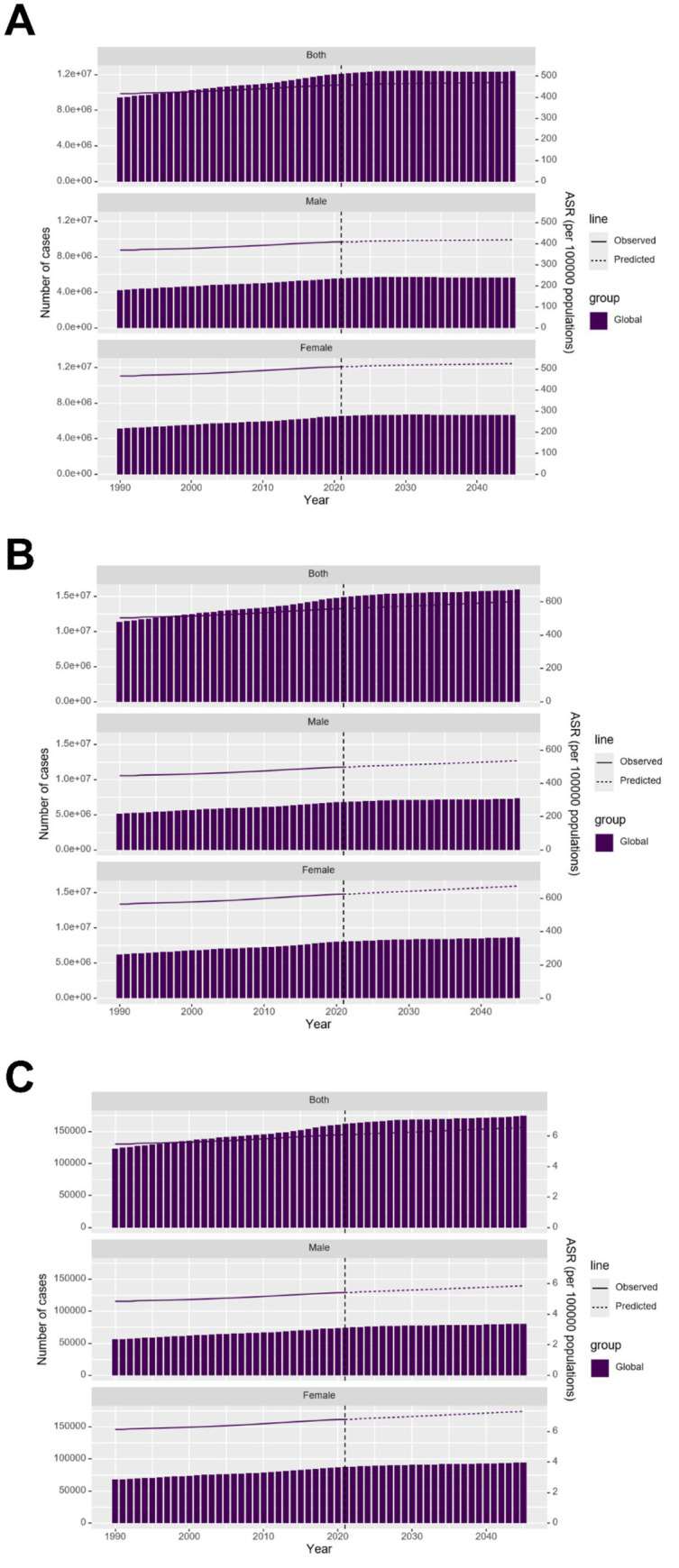
Projected age-standardized rate of **(A)** incidence, **(B)** prevalence, and **(C)** DALYs attributable to pruritus by sex based on the BAPC model.

Using the INLA framework and BAPC methodology, the global pruritus burden up to 2045 was further predicted (Figure 7). At the global scale, the projections indicated an increase in pruritus metrics by 2045, with the ASIR, ASPR, and ASDR expected to be up to 470.3 per 100,000 population (95% CI, 210.52 to 730.08), 602.79 per 100,000 population (95% CI, 272.36 to 933.23), and 6.56 per 100,000 population (95% CI, 2.8 to 10.33), respectively (Table 4). Specifically, compared to 2021, the ASIR of newly reported pruritus is anticipated to increase by over 2%, and the corresponding ASPR and ASDR are projected to grow by over 7% up to 2045. Additionally, our projections also indicated that sex disparities would remain over the same period, namely, higher ASIR, ASPR, and ASDR in females than in male children and adolescents (Supplementary Table 5).

**Table 4:** The prediction of ASIR, ASPR, and ASDR attributable to pruritus in the world by 2045 (per 100,000).

Gender	ASIR (95% CI)	ASPR (95% CI)	ASDR (95% CI)
Both	470.3 (210.52 to 730.08)	602.79 (272.36 to 933.23)	6.56 (2.8 to 10.33)
Female	525.47 (236.08 to 814.86)	673.99 (304.59 to 1043.38)	7.32 (3.01 to 11.63)
Male	418.52 (186.04 to 651)	535.95 (241.83 to 830.06)	5.86 (2.38 to 9.35)

## 4 Discussion

Clinically, pruritus can be classified as acute, chronic (lasting for more than 6 weeks), or acute attacks during a chronic course. Theodosiou et al. recently showed that 20.0 and 14.7% of the 443 German schoolchildren are suffering from acute and chronic pruritus, respectively (15). Halvorsen et al. showed that 8.8% of the late adolescents in Norway complain about being troubled by pruritus and proved the positive association between pruritus and mental distress (16). Dalgard et al. also presented similar findings among 18-year-old boys and girls from Boston (17). As Kang et al. (18) summarized, many dermatoses (including atopic dermatitis, acne vulgaris, seborrheic dermatitis, contact dermatitis, psoriasis, and urticaria) in children and adolescents cause pruritus, and the study on atopic dermatitis-related pruritus is the most extensive since atopic dermatitis brings the heaviest disease burden and is currently a difficult clinical problem to solve in dermatology (19). However, the incidence of pruritus varies significantly among patients without dermatosis. Approximately 40–90% of hemodialysis patients, 30% of patients with Hodgkin lymphoma, 15% of patients with non-Hodgkin lymphoma, 67% of patients with polycythemia vera, 13.6% of males and 7.4% of females with iron-deficiency anemia, and 13–45% of HIV-positive patients experience varying degrees and types of pruritus (5). According to a meta-analysis of all-grade pruritus in cancer patients treated with biological therapies, panitumumab (56.8%) and gefitinib (49.4%) cause the highest incidence of pruritus, while erlotinib (3.6%) and sunitinib (5.8%) cause the lowest incidence (20). Another meta-analysis shows that about 52% of maintenance hemodialysis patients in China complain about pruritus, with 26, 22, and 8% experiencing mild, moderate, and severe degrees, respectively (21).

Rigorous, large-scale, global, cross-sectional, and population-based surveys on the epidemiology of pruritus in children and adolescents are currently lacking. This study focused on evaluating the pruritus burden in children and adolescents aged under 20 years from 1990 to 2021 based on the pre-existing GBD 2021 database. This database is widely recognized by public health experts and has been used thousands of times for reanalysis according to different topics. Regarding the pruritus burden in children and adolescents aged under 20 years, we showed increasing ASIR, ASPR, and ASDR of worldwide pruritus from 1991 to 2021, indicating a gradually increasing burden on healthcare. However, in clinical practice, pruritus has not received sufficient attention, especially in non-dermatology departments. The fact that non-lethal pruritus is not an independent disease but a symptom that accompanies many diseases may contribute to its undervaluation. Dermatologists attach great importance to pruritus because pruritic diseases are the main diseases they currently face, and they will try their best to explore the pathogenesis and treatment strategies for pruritus in different disease states. Pruritus experts in Europe and China have developed updated guidelines on chronic pruritus (22, 23), and pediatric dermatologists in China have also established an expert consensus on chronic pruritus derived from dermatosis in children (24). They proposed that it is imperative to collect high-quality epidemiological data on pruritus in various populations.

Based on the GBD 2021 database, Jin et al. demonstrated 9.83, 10.33, and 10.26% increases in the global ASIR, ASPR, and ASDR, respectively, for pruritus among the whole population without age consideration (7). The current study, focusing on children and adolescents aged under 20 years, also showed an increased pruritus burden over time, with an ASIR of 458.02, ASPR of 559.72, and ASDR of 6.09 in 2021. We further found higher pruritus-related ASIR, ASPR, and ASDR in female children and adolescents than in male children and adolescents, consistent with the findings observed in all populations from the analysis conducted by Wang et al. (6). The differences between genders in children and adolescents may be associated with the fact that some pruritic diseases occur more frequently in female children and adolescents, such as atopic dermatitis (25), lichen planus (26), autoimmune urticaria (27, 28), and psychosocial disorders (29, 30). This is different from the elderly women reporting more pruritus symptoms due to hormonal changes and immune senescence (31). Interestingly, we, along with other researchers, have shown that the incidence, prevalence, and DALYs of pruritus gradually increase as age increases in children and adolescents (6, 7), which is inconsistent with the analysis results in the adult and elderly populations, where more diverse changes are exhibited. The steady increase in burden with age may be due to the onset of specific conditions, such as acne vulgaris or atopic dermatitis in adolescence (32, 33), and changes in contact allergen exposure may also be one of the reasons (34, 35).

Using the BAPC model under the INLA framework, we projected the pruritus burden in children and adolescents by 2045 and found that there may be a total of 12385010.06 new cases (6707193.11 in females and 5679967.88 in males); correspondingly, the global ASPR of pruritus in children and adolescents may increase from 559.72 to 602.79, that from 625.2 to 673.99 in females, and that from 498.12 to 535.95 in males. Decomposition analysis in female and/or male children and adolescents confirmed that population growth, but not epidemiological changes and aging, is the key driving force behind the increased burden of pruritus. In this regard, we should note that the forecast is based on the trend of population growth, but with the current slowdown in the growth rate of newborns (at least in China) (36, 37), new cases of pruritus in children and adolescents by 2045 may decrease. However, in clinical practice, with the improvement of disease monitoring networks and the convenience of seeking medical treatment, more cases from the general population complaining of itching or increasing pruritus in diseases with the widespread application of biologics may be recorded, which may not depend on population growth.

Our study also found the highest pruritus-related ASIR, ASPR, and ASDR in children and adolescents in countries with a low SDI, which is partially consistent with the distribution of pruritus burden in regions, countries, or nations. Specifically, Eastern/Southern/Western Sub-Saharan Africa and the United Republic of Tanzania have the heaviest pruritus burden among children and adolescents, while Germany has the lightest burden. Changes in infectious etiologies and allergy exposure in different SDI regions may contribute to differences in pruritus burden. In addition, the growth rate of the pruritus burden should be particularly noted. The most rapid escalation in incidence was found in East Asia and the Netherlands, whereas the most significant increase in prevalence and DALYs was found in Australasia in our analysis.

This study has several limitations. First, when the pruritus was recorded in the GBD 2021 database, the triggering factors or underlying diseases that cause pruritus were not also recorded, which makes it difficult for us to accurately analyze the incidence, prevalence, and DALYs of pruritus in different disease types in children and adolescents. Second, because of the lack of sufficient information, we failed to calculate the burden of acute and chronic pruritus in children and adolescents separately. Third, as with all reanalyses based on the GBD databases, the accuracy of the existing raw data will, to some extent, influence our conclusions. Future studies should prospectively collect detailed clinical data on pruritus characteristics, including duration (acute vs. chronic), intensity (e.g., using visual analog scales), and associated dermatological or systemic diagnoses. Of course, the existence of potential confounding factors, including differences in regional economic levels and health policies, warrants further investigation based on real-world populations using a unified research protocol, especially a standardized pruritus reporting strategy.

## 5 Conclusion

In summary, the overall burden of pruritus in children and adolescents has risen substantially from 1990 to 2021 and increases with age, with females and individuals in low-SDI regions, including Eastern/Southern/Western Sub-Saharan Africa and the United Republic of Tanzania, being more affected. The burden may gradually increase with population growth in the next 20 years. Further strengthening policies to tackle burdensome pruritus is warranted, especially in East Asia, Australasia, and the Netherlands, where the burden of pruritus in children and adolescents is increasing rapidly. Specifically, infectious disease control and primary care access may be the main areas to be strengthened in low-SDI regions with a high burden, while allergy prevention or mental health co-management should be focused on in high-SDI regions with rapid increases.

## Data Availability

The original contributions presented in the study are included in the article/Supplementary material, further inquiries can be directed to the corresponding authors.

## Footnotes

1
https://vizhub.healthdata.org/gbd-results/
